# Malaria epidemiology in Kobeni department, southeastern Mauritania from 2015 to 2017

**DOI:** 10.1186/s40249-020-0634-5

**Published:** 2020-02-12

**Authors:** Sileye Mamadou Diallo, Hervé Bogreau, Nasserdine Papa Mze, Mohamed Salem Ould Ahmedou Salem, Mohamed Lemine Ould Khairy, Philippe Parola, Leonardo Basco, Ali Ould Mohamed Salem Boukhary

**Affiliations:** 1 Unité de Recherche Génomes et milieux (Jeune Equipe Associée à l’Institut de Recherche pour le Développement [IRD]), Faculté des Sciences et Techniques, Université de Nouakchott Al-Aasriya, Nouakchott, Mauritania; 2 Aix Marseille Université, IRD, Assistance Publique-Hôpitaux de Marseille (AP-HM), Service de Santé des Armées (SSA), Vecteurs – Infections Tropicales et Méditerranéennes (VITROME), Marseille, France; 3 Institut Hospitalo-Universitaire (IHU)-Méditerranée Infection, Marseille, France; 4 Unité Parasitologie et Entomologie, Département des maladies infectieuses, Institut de Recherche Biomédicale des Armées, Marseille, France; 5 Ministère de la Santé, Programme National de Lutte contre le Paludisme (PNLP), Nouakchott, Mauritania; 6 Present Address: Direction régionale de l’action sanitaire, Nouakchott sud, Ministère de la santé, Nouakchott, Mauritania

**Keywords:** *Plasmodium falciparum*, *Plasmodium vivax*, Drug resistance, Cross-border malaria, Epidemiology, Sahel

## Abstract

**Background:**

*Plasmodium falciparum* malaria is endemic in the southern sahelian zone of Mauritania where intense internal and trans-border human and livestock movement occurs. The risk of importation and spread of drug-resistant parasites need to be regularly assessed in this region. The objective of the study was to assess the recent malaria situation near the Mauritania-Mali border.

**Methods:**

Between February 2015 and December 2017, patients with fever or history of fever during the previous 48 h, presenting at the health centre of Kobeni city, were screened for malaria using a rapid diagnostic test (RDT) and microscopic examination of blood smears. The diagnosis was later confirmed by PCR. Cohen’s kappa statistics was used to estimate the degree of agreement between diagnostic methods. Fisher’s exact test was used to compare proportions. The odds ratio was calculated to measure the association between the use of bed nets and malaria infection.

**Results:**

A total of 2326 febrile patients (mean age, 20.2 years) were screened for malaria. The presence of malaria parasites was detected by RDT and microscopy in 53.0% and 49.3% of febrile patients, respectively, and was confirmed by PCR in 59.7% (45 missing data). Of 1361 PCR-positive samples, 1205 (88.5%) were *P. falciparum*, 47 (3.5%) *P. vivax*, and 99 (7.3%) *P. falciparum-P. vivax* mixed infection*.* Malaria transmission occurred mostly during and shortly after the rainy season. The annual rainfall was relatively low in 2016 (267 mm) and 2017 (274 mm), compared to 2015 (448 mm), and coincided with a decline in malaria prevalence in 2016–2017. Although 71.8% of febrile patients reported to possess at least one bed net in the household in our questionnaire, its reported use was not protective against malaria infection (odds ratio: 1.1, 95% *CI*: 0.91–1.32).

**Conclusions:**

Our study confirmed that *P. falciparum* is the dominant species in the sahelian zone and that malaria transmission is seasonal and associated with rainfall in this zone. The application of the current national policy based on rapid and reliable malaria diagnosis, case management with artemisinin-based combination therapy, intermittent preventive treatment for pregnant women, distribution and use of long-lasting insecticide impregnated bed nets, and the planned introduction of seasonal malaria chemoprevention for all children under 6 years old is expected to sustainably reduce malaria transmission in this zone.

## Background

Despite decades of control efforts, *Plasmodium falciparum* malaria is still endemic in southern sahelian zone of Mauritania, as well as in parts of northern Saharan zone where *Plasmodium vivax* is more common [1–3]. Cases infected with *Plasmodium malariae* and *Plasmodium ovale* have rarely been reported in the country [2, 4]. Overall, malaria transmission is seasonal with sporadic outbreaks in Mauritania. *Anopheles gambiae* sensu lato (s.l.) is the main malaria vector in the country [5–7]. Pyrethroid resistance in *Anopheles arabiensis* has recently been reported in the Saharan and sahelian zones [8, 9].

Nomadic life and internal and trans-border human (i.e., nomadism) and livestock (i.e., transhumance) movement have long been the hallmarks of the sahelian region [10]. Population movement has intensified in recent years due to dynamic regional commercial exchanges, reinforced interdependence between neighboring countries, and construction of paved roads [11]. Moreover, the political instability in Mali, Mauritania’s neighboring country to the south, and the resulting inter-ethnic conflicts have led to mass influx of Malian refugees into southern Mauritania [12, 13]. The sahelian zone, particularly Hodh Elgharbi region, has also become a transit zone for thousands of migrants each year from sub-Saharan Africa on their way to Europe [11, 13]. Population movements increase the risk of importation and spread of malaria parasites, including drug-resistant strains, compromising control efforts and programmes. Because of its longstanding malaria endemicity and proximity to border areas with on-going malaria transmission, Kobeni department in Hodh Elgharbi province could potentially be at the heart of malaria transmission ‘hotspots’ where disease surveillance needs to be reinforced. Moreover, a better understanding of the dynamics of malaria transmission in this region has become a necessity to ensure malaria containment and elimination from the region. The objective of the present study was to assess and update data on malaria burden and *Plasmodium* species in Kobeni city, the largest urban city in the sahelian zone near the border with Mali.

## Methods

### Study area

The sahelian region of Mauritania consists of an east-to-west belt of steppes and savannah grasslands lying between 150 mm isohyets to the North (i.e., the southern limit of the Saharan desert) and 500 mm isohyets to the South (i.e., the northern limit of the Sudanian savannah). It occupies 11% of the total surface area of Mauritania but has about 61% of the total population distributed across seven administrative provinces: Trarza, Brakna, Gorgol, Assaba, Guidimagha, Hodh Elgharbi and Hodh Echarghi [14]. While the population density of the northern Saharan part of Mauritania is about 2.2 persons/km^2^, that of the sahelian zone is more than 6 persons/km^2^.

**Fig. 1:**
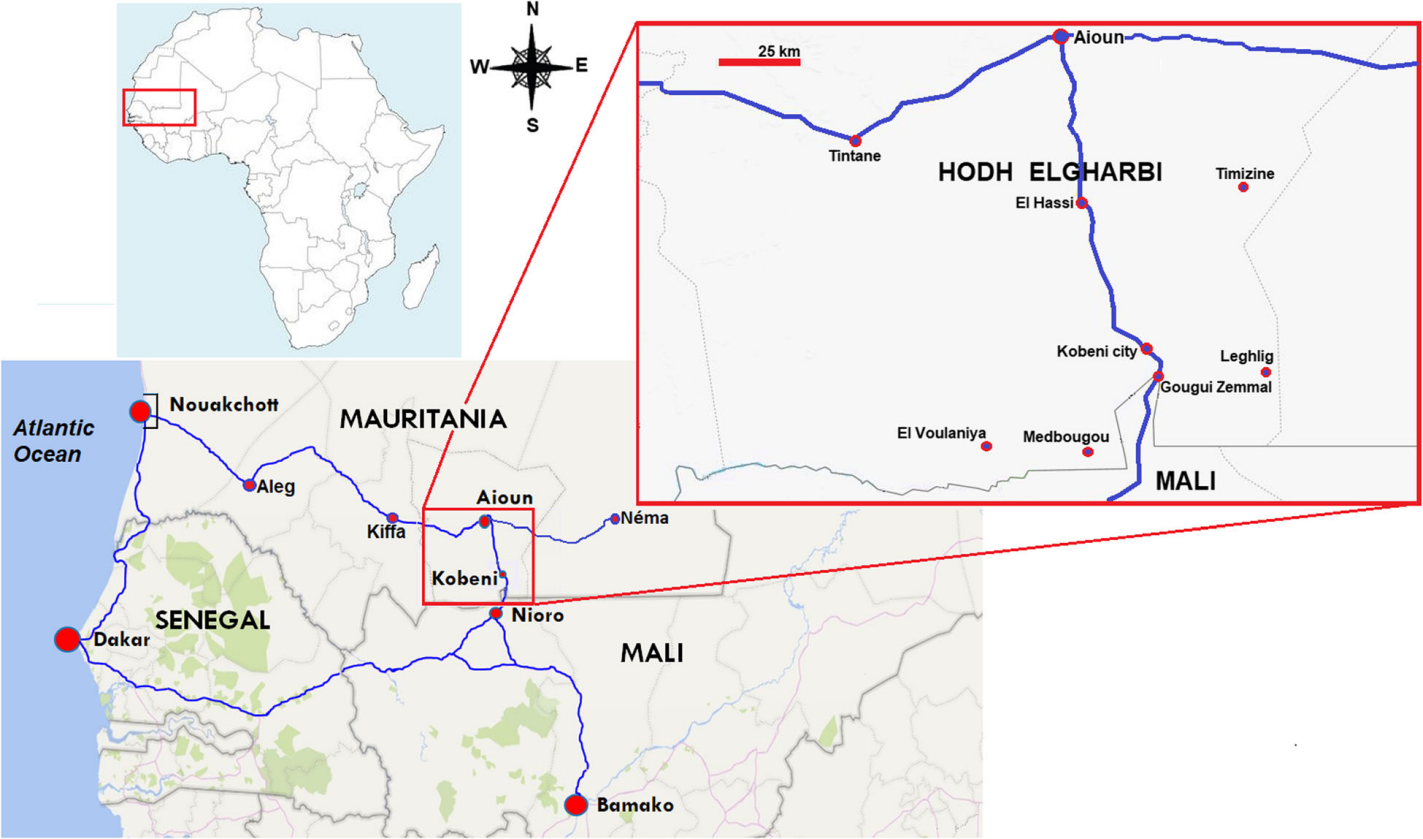
Map showing the location of Kobeni city. The blue line indicates paved roads. The road connecting Nouakchott and Aioun is part of ‘Route de l’Espoir’ which continues eastward from Aioun and ends at the city of Néma in southeastern Mauritania

Our study was conducted between 2015 and 2017 in the city of Kobeni (15°49′N; 9°24′W; altitude above sea level, 200 m), an urban city with agropastoral activities, situated at about 18 km from the border with Mali (Fig. 1). The city of Kobeni is the capital of Kobeni department, one of the four departments of the administrative province of Hodh Elgharbi. Kobeni department is comprised of seven communes, namely El Hassi, Gougui Zemmal, Kobeni, Leghlig, Medbougou, Timizine and El Voulaniya, with a total of 92 690 inhabitants among whom 11 833 reside in Kobeni city [15, 16]. Kobeni is at the cross-roads between southeastern Mauritania and Mali. The only existing paved road passing through Kobeni city connects Nouakchott, the capital city of Mauritania, via the ‘route de l’Espoir’, and Bamako, in Mali, and bifurcates from the town of Nioro, situated near the border with Mauritania, to reach Dakar, Senegal.

The population in Kobeni is mainly composed of Moors, an ethnolinguistic groups that speak a Berber-influenced Arabic language, and people of black African origin (Pular, also known as Fulani, and Soninke). There are also some minorities belonging to Bambara (the major ethnic group in Mali) and Tuareg ethnic groups. Approximately 49% of the inhabitants of Kobeni department are living below the poverty line (i.e. less than 1.9 dollars a day) [16]. Subsistence farming and livestock production are the main economic activities in Kobeni department.

The climate in Kobeni is of the sahelian type, characterized by a long dry season from October to June and a short wet season from July to September. The amount of rainfall and its spatial distribution are irregular between years and sometimes even during the same year [17]. Annual rainfall during the study period was 448, 267, and 263 mm in 2015, 2016 and 2017, respectively (National Office of Meteorology, unpublished data). Temperature data in Kobeni city have not been recorded by the local weather station. The closest meteorological station located in Aioun city, 100 km to the north of Kobeni, recorded a mean annual temperature of 29.7 °C (range: 23.6–35.8 °C) and the months of May and June as the hottest months [16].

During the study period, the health centre of Kobeni city was the only secondary public health structure in the entire Kobeni department. The medical staff included one general medical practitioner, four nurses, and one laboratory technician. In each commune, there is also a primary health care centre. The nearest public health centre outside Kobeni city is located in Aioun, about 100 km to the north of the city. This fact largely explains why patients from several localities outside Kobeni city come to the health centre in Kobeni city for medical consultation, particularly during the malaria transmission season.

### Study population, inclusion criteria, and sample collection

The study population consisted of patients of all ages consulting the health centre of Kobeni from February 2015 to December 2017. The inclusion criteria of “malaria suspected cases” were fever (a measured axillary temperature of ≥ 37.5 °C) at the time of medical consultation or history of fever within 48 h before consultation, with clinical syndrome suggesting malaria [18]. After obtaining informed consent from the patient (or from the accompanying parents or legal guardians for children), finger-prick capillary blood samples were obtained to prepare thick and thin smears and perform rapid diagnostic test (RDT) for malaria. Capillary blood samples (100 μl) were spotted on Whatman 3MM filter paper (GE Healthcare Europe GmbH, Vélizy Villacoublay, France), dried and stored for molecular analysis. During consultation, the patients or the parents (or legal guardians) of febrile children were interviewed to obtain socio-demographic data, including origin, internal and outside recent travel history, and bed net use, and their responses were recorded using a standard, pretested questionnaire.

### Rapid diagnostic test

The Standard Diagnostics (SD) Bioline malaria antigen Pf/Pan test (Standard Diagnostics/Abbott, https://www.abbott.com) was used according to the manufacturer’s instructions. This RDT detects *P. falciparum*-specific histidine-rich protein 2 (PfHRP-2) and *Plasmodium* genus-specific lactate dehydrogenase (pLDH). RDT results were blinded with regards to microscopic examination and PCR results.

### Microscopy

Thick and thin blood films were prepared, stained with 5% Giemsa solution for 20 min, and examined for the presence of malaria parasites. Thick blood film was considered as negative if no asexual stage of *Plasmodium* spp. was found after an examination of 100 fields under oil immersion at a magnification of × 1000. Parasite density was determined by counting the number of asexual parasites per 200 leukocytes and assuming a leukocyte count of 8000/μl of blood [19]. The final parasite density was recorded as the average count of two experienced technicians. An experienced microscopist performed quality control by reexamining all positive samples and 10% of negative blood smears blindly.

### Polymerase chain reaction (PCR)

Parasite DNA was extracted from dried blood spots using an automated nucleic acid purification system (MagMAX™-Express, Thermo Fisher Scientific, Montigny-le-Bretonneux, France) following the manufacturer’s instructions. *Plasmodium* species was identified using the PCR protocol developed by Snounou et al. (1993) with fluorescent-dye-labeled oligonucleotides [20]. The PCR products were analyzed by capillary electrophoresis.

### Statistical analysis

Data were entered into an Excel spreadsheet (Microsoft Office Excel 2007, Microsoft Corporation, Redmond, WA, USA). Fisher’s exact test was used to compare proportions and test association between qualitative variables. Cohen’s kappa statistics was used to estimate the degree of agreement between diagnostic methods [21–23]. The kappa coefficient was classified as follows: < 0, no agreement; 0–0.20, none to slight; 0.21–0.40, fair; 0.41–0.60, moderate; 0.61–0.80, substantial; and > 0.80, almost perfect agreement. The odd ratios were computed according to Altman (1991) using MedCalc statistical software (MedCalc Software, Ostend, Belgium) [23]. For all statistical tests, the significance level was set at *P*-value < 0.05.

## Results

### Patient characteristics

Overall, 2326 febrile patients (*n* = 1247 in 2015, *n* = 566 in 2016, *n* = 513 in 2017), mostly composed of Moorish population (87.7%, 2040/2326), were recruited (Table 1). Other ethno-linguistic groups (Pular, Soninke, Wolof, and Bambara), collectively also known as ‘Black Africans,’ constituted 12.3% (286/2326) of the patient population. The proportion of females (53.2%; 1238/2326) was significantly higher (*P* = 0.002, 95% *CI*: 2.3–10.4%) than males (46.8%, 1088/2326) with a female-to-male ratio of 1.14. The mean age was 20.2 years (range, 1 month old to 87 years old), and the median age was 17 years. Children under 5 years old represented 11.1% (259/2326) of the study population. The mean (± standard deviation) axillary temperature at the time of consultation was 38.7 °C (± 1.1 °C).

**Table 1:** Characteristics of febrile and malaria-infected patients consulting at Kobeni health centre in Hodh Elgharbi region, southeastern Mauritania, during 2015–2017

Characteristics	Study year			
	2015	2016	2017	2015–2017
Number	1247	566	513	2326
Gender				
Female, *n* (%)	640 (51.3)	311 (54.9)	287 (56.0)	1238 (53.2)
Male, *n* (%)	607 (48.7)	255 (45.1)	226 (44.0)	1088 (46.8)
Age group (year)				
< 5, *n* (%)	138 (11.1)	77 (13.6)	44 (8.6)	259 (11.1)
5–9, *n* (%)	192 (15.4)	103 (18.2)	67 (13.1)	362 (15.6)
10–14, *n* (%)	175 (14.0)	82 (14.5)	68 (13.2)	325 (14.0)
≥ 15, *n* (%)	742 (59.5)	304 (53.7)	334 (65.1)	1380 (59.3)
Mean axillary temperature (range) (°C)	38.7 (37.5–40.9)	38.8 (37.5–40.5)	38.6 (37.5–40.9)	38.7 (37.5–40.9)
GMPD^a^ (asexual parasites/μl)	1990	1860	2640	1100
PCR-positive by age group (years)				
< 5, *n* (%)	71 (9.0)	32 (11.2)	22 (7.6)	125 (9.2)
5–9, *n* (%)	138 (17.6)	53 (18.5)	39 (13.5)	230 (16.9)
10–14, *n* (%)	113 (14.4)	43 (15.0)	39 (13.5)	195 (14.3)
≥ 15, *n* (%)	464 (59.0)	158 (55.2)	189 (65.4)	811 (59.6)
Ethno-linguistic groups				
Moors^b^*n* (%)	1101 (88.3)	502 (88.6)	437 (85.1)	2040 (87.7)
Black Africans^c^*n* (%)	146 (11.7)	64 (11.4)	76 (14.9)	286 (12.3)

^a^Geometric mean of parasite density

^b^Moors refer to ethno-linguistic group speaking *Hassaniya*, a Berber-influenced Arabic dialect

^c^Black Africans refer to ethnic groups (Pular [also known as Fulan], Soninke, Wolof and Bambara [the main ethnic group in Mali])

### Malaria prevalence and parasite species

RDT and microscopy were performed on all recruited patients (*n* = 2326). However, dried blood spots were available for PCR in 2281 (98.1%) patients. The prevalence of malaria by RDT, microscopy, and PCR was 53.0% (1232/2326), 49.3% (1146/2326), and 59.7% (1361/2281), respectively. Among 1361 PCR-positive cases, 1205 (88.5%) were due to *P. falciparum*, 47 (3.5%) *P. vivax*, and 99 (7.3%) *P. falciparum*-*P. vivax* mixed infections (Table 2). Few cases (*n* = 10; 0.7%) of *P. malariae*, either alone or mixed infections, were also observed among PCR-positive samples. The RDT used in the present study did not make a distinction between *P. falciparum* alone and *P. falciparum* with another *Plasmodium* species when the ‘Pan’ band was visible. Using microscopy, almost all smear-positive cases (99.7%; 1143/1146) were due to *P. falciparum*. The remaining three positive blood samples were diagnosed as *P. ovale* (0.2%) or *P. malariae* (0.1%). Parasite density varied between 42 to 482 000 asexual parasites/μl of blood, with a geometric mean of 1100 asexual parasites/μl of blood. Adults (arbitrarily defined as > 15 years) were the most affected (59.6%), followed by the age group of 5–9 years old (16.9%; *P* < 0.05). Children less than 5 years old were the least affected (9.2%; *P* < 0.0001) (Table 1).

**Table 2:** Proportions of malaria-positive tests by diagnostic methods among febrile patients consulting at Kobeni health centre in Hodh Elgharbi region, south-eastern Mauritania, during 2015–2017^a^

*Plasmodium* spp.	Number (%) of patients	
	Microscopy	PCR^b^
*P. falciparum* (*Pf*)	1143 (99.7)	1205 (88.5)
*P. vivax* (*Pv*)	0	47 (3.5)
*P. ovale*	2 (0.2)	0
*P. malariae* (*Pm*)	1 (0.1)	3 (0.2)
*Pf-Pv*	0	99 (7.3)
*Pf-Pm*	0	6 (0.4)
*Pf-Pv-Pm*	0	1 (0.07)

^a^Results are expressed as the number of malaria-positive samples and the proportions of malaria species among 1146/2326 (49.3%) samples that were positive by microscopic examination and 1361/2281 (59.7%) samples that were positive by PCR

^b^PCR was not performed in 45 febrile patients due to the absence of dried blood spots. RDT was positive for *P. falciparum*, with or without other *Plasmodium* spp., in 1232/2326 (53.0%)

RDT and microscopy results exhibited a substantial agreement (Cohen’s kappa coefficient: 0.8; 95% confidence interval [*CI*]: 0.77–0.82). However, the comparison of these two diagnostic methods with PCR yielded only a fair agreement between RDT and PCR (Cohen’s kappa coefficient: 0.34; 95% *CI*: 0.30–0.38) and between microscopy and PCR (Cohen’s kappa coefficient: 0.30; 95% *CI*: 0.27–0.34).

### Malaria infection and rainfall patterns

**Fig. 2:**
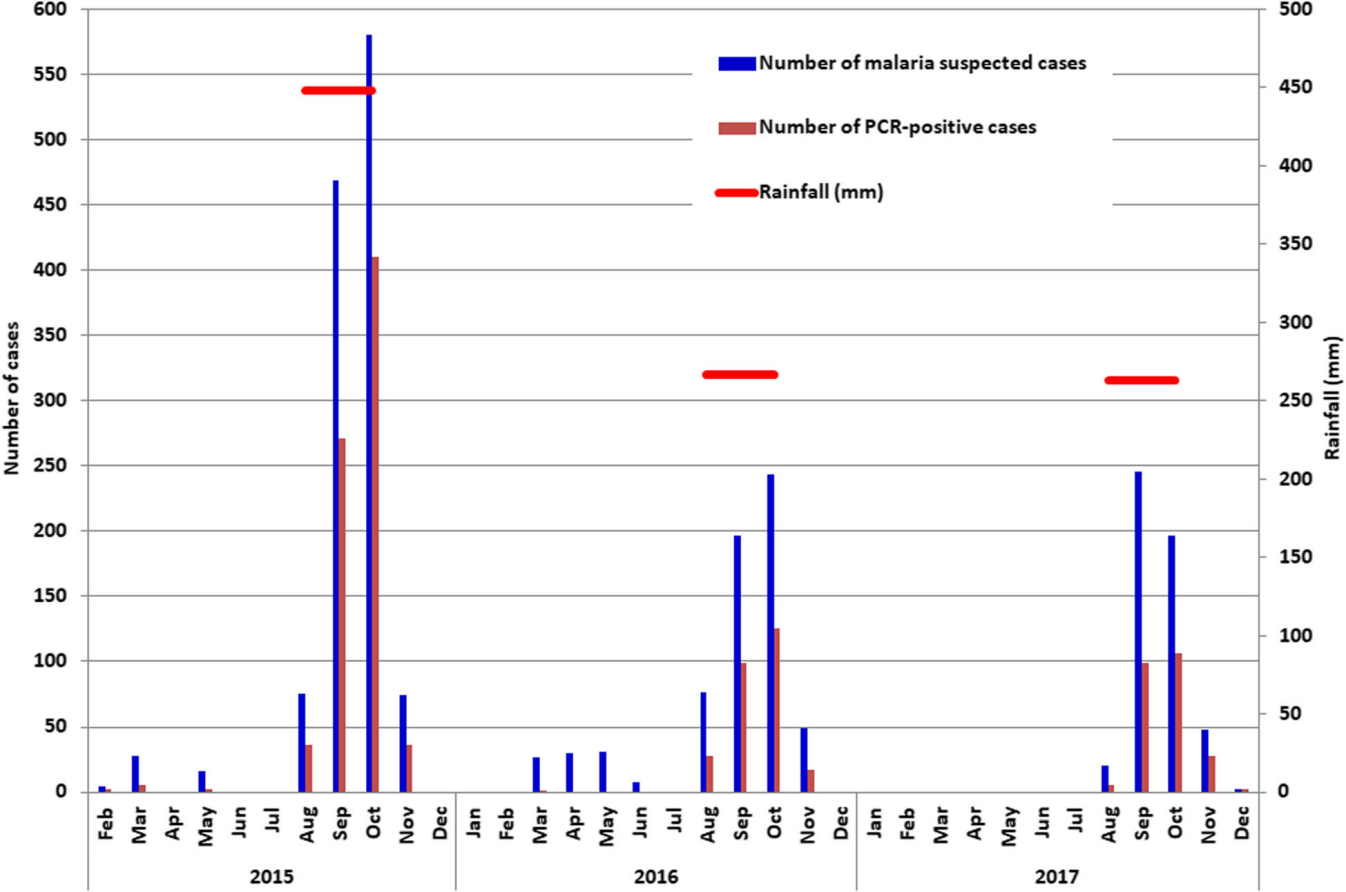
The number of presumptive malaria cases among febrile patients and number of PCR-positive cases at Kobeni health centre in 2015–2017. The red horizontal bars denote the total annual rainfall and the duration of rainy season for each year. Patients were not recruited during the dry season (January–July) in 2017

During the study period, malaria infections occurred mainly between August and November, with a peak in September or October, corresponding to the unimodal rainfall pattern that characterizes the sahelian climate (July–September) (Fig. 2). During the dry seasons of 2015 and 2016, only 10 (0.94%) malaria cases were diagnosed by PCR. In 2017, patient recruitment was suspended between January and June because of the very low number of malaria cases seen in the two preceding years. The amount of rainfall decreased from 448 mm in 2015 to 267 mm in 2016 and 263 mm in 2017, which coincided with a decline in malaria transmission during the peak season in both 2016 and 2017.

### Origin and travel history of malaria-infected patients

Of 2281 febrile patients for whom PCR was performed, 746 (32.7%) came from outside the commune of Kobeni (Table 3). These patients either were residents of one of the six rural communes of the Department of Kobeni or came from another sub-Saharan African country (Mali, Senegal, Côte d’Ivoire, or Republic of Congo). Based on PCR results, malaria prevalence was 66.1% (899/1361) among the residents of Kobeni commune, and 33.9% (462/1361) among those residing outside Kobeni commune.

**Table 3:** Origin, travel history, and malaria positivity among febrile patients consulting at Kobeni health centre in Hodh Elgharbi region, south-eastern Mauritania, during 2015–2017

	Number (%) of patients^a^	
	Febrile	PCR-positive
Origin	*n =* 2281	*n =* 1361
From Kobeni commune	1535 (67.3)	899 (66.1)
From outside Kobeni	746 (32.7)	462 (33.9)
Travel history	*n =* 102	*n =* 53
External travel^b^	31 (30.4)	18 (34.0)
Internal travel^c^	71 (69.6)	35 (66.0)
No travel history outside Kobeni department (commune of residence)	*n =* 2179	*n =* 1308
Kobeni city	1464 (67.3)^d^	864 (59.0)^e^
Gougui Zemmal	168 (7.7)^d^	102 (60.7)^e^
El Hassi	245 (11.2)^d^	150 (61.2)^e^
Medbougou	221 (10.1)^d^	147 (66.5)^e^
Leghlig	29 (1.3)^d^	13 (44.8)^e^
El Voulaniya	46 (2.1)^d^	29 (63.0)^e^
Timizine	6 (0.3)^d^	3 (50.0)^e^

^a^PCR was performed in 2281 febrile patients

^b^From Côte d’Ivoire, Republic of Congo, Mali, or Senegal (3/18 of PCR-confirmed malaria patients were Malians arriving from Mali)

^c^internal travel refers to patients who had travelled to malaria endemic zones in southern Mauritania or to the northern zone where *P. vivax* is known to be endemic (Nouakchott and Atar)

^d^Percentages of febrile cases without any travel history in each commune (denominator, 2179)

^e^Malaria infection rate in each commune, defined as the number of PCR-positive malaria cases among febrile cases in each commune

Among 102 febrile patients with a recent travel history, 31 (30.4%) declared to have travelled to another malaria endemic country in sub-Saharan Africa, and 71 (69.6%) reported to have travelled to areas in Mauritania known to be endemic for malaria, i.e. zones in southern Mauritania, Nouakchott, or Atar in northern Mauritania. Among these febrile patients with a travel history to endemic areas, 53 (52%) had PCR-confirmed malaria, including three Malians arriving from their country. Febrile patients without any recent travel history (*n* = 2179) seeking care at Kobeni health centre were residents in one of the seven communes of the Department of Kobeni. Among these local residents, 1308 (60.0%) were PCR-positive. No significant difference was found between malaria prevalence among patients with travel history and those with no travel history (*P* = 0.24).

### Association between bed net use and malaria infection

Information on bed net use was obtained from 2281 of 2326 (98.1%) febrile patients, among whom 1358 (59.5%) had PCR-confirmed malaria. The proportion of patients who reported to own and use bed nets, either treated or untreated with insecticides (1637/2281, 71.8%), was significantly higher (*P* < 0.0001; 95% *CI*: 39.3–47.5%) than those who reported that they do not own or use bed nets (28.9%) (Table 4). Residents who completed their primary education or went to a Koranic school tended to own and use bed nets more than those who did not complete their primary education (82% vs 69%; *P* < 0.05). However, the reported use of either insecticide-treated or insecticide-untreated bed nets did not reduce the probability of being infected by malaria (odds ratio: 1.1, 95% *CI*: 0.91–1.32), as compared to non-use of bed nets (*P* > 0.05).

**Table 4:** Bed net use and malaria infections among febrile patient consulting at Kobeni health centre in Hodh Elgharbi region, southeastern Mauritania, during 2015–2017

Bed net use^a^	*N* (%)	Number (%) of patients			
		Malaria positive	Malaria negative	Odd ratio (95% *CI*)	*P*-value
Yes	1637 (71.8)	987 (60.3)	650 (39.7)	1.1 (0.91–1.32)	0.33
No	644 (28.2)	374 (58.1)	270 (41.9)		

^a^Information on the use of bed net was available from 2281/2326 (98.1%) patients. Among those who own bed nets, a large majority of patients reported to have both insecticide-impregnated and non-impregnated bed nets

### Clinical manifestation and malaria treatment practice

Of 1232 RDT-confirmed malaria infected patients during 2015–2017, 151 (12.2%) presented signs and symptoms suggestive of severe malaria: multiple convulsions, lethargy, anuria, jaundice, dark-colored urine, and palmar pallor. The other patients (1081; 87.7%) presented signs and symptoms of uncomplicated malaria. Quinine salts were the most frequently used antimalarial drug in the health centre of Kobeni to treat confirmed malaria cases, regardless of the clinical manifestations, i.e. either severe or uncomplicated malaria (Table 5). Artesunate-amodiaquine or artemether-lumefantrine, antimalarial drugs recommended by the Mauritanian Malaria Control Unit and the WHO to treat uncomplicated malaria, was infrequently used during the study period.

**Table 5:** Antimalarials used to treat malaria infections in patient consulting at Kobeni health center in Hodh Elgharbi region, southeastern Mauritania, during 2015–2017

Year	*N* (%)					
	Quinine	ASAQ	AL	SP	CQ	AS-SP
2015	703 (64.4)	23 (1.8)	2 (0.2)	27 (2.2)	31 (2.5)	17 (1.4)
2016	296 (52.3)	0	1 (0.2)	0	7 (1.2)	3 (0.5)
2017	284 (55.3)	0	0	0	0	0
Total	1283 (55.2)	23 (1)	3 (0.1)	27 (1.2)	38 (1.6)	65 (0.9)

*ASAQ* Artesunate-amodiaquine, *AL* Artesunate-lumefantrine, *SP* Sulfadoxine-pyrimethamine, *CQ* Chloroquine, *AS-SP* Artesunate-sulfamethoxypyrazine-pyrimethamine

## Discussion

Kobeni is the only sentinel site in Mauritania where studies on malaria epidemiology have been conducted regularly by several research teams over the past 20 years [2, 7, 9, 24–33]. Kobeni is situated within the ‘heart’ of area of seasonal but intense malaria transmission in Mauritania. Previous parasitological studies in Kobeni were conducted in a limited number of symptomatic children and adults (*n* = 663) in 2009–2010 [2] and asymptomatic children (*n* = 345) in 2011–2013 [32]. The present study was carried out to update parasitological data in a larger sample (*n* = 2326) of patient population over three consecutive years. The results of the present study showed that 49% (1146 of 2326) of febrile patients had microscopy-confirmed malaria, mostly due to *P. falciparum* (99.7%). These results confirm the data of earlier parasitological studies based on microscopy conducted in 2009–2010 in Kobeni in which 57% (378/663) of febrile patients had microscopy-confirmed malaria, of which 96.6% were due to *P. falciparum* [2]. RDT was introduced in the country in 2010, and PCR was not performed in earlier studies in Kobeni. The comparison of microscopy data in these two studies conducted under similar conditions at the same health centre implies that malaria prevalence remained stable in Kobeni between 2009 and 2017.

The majority of febrile patients and PCR-positive malaria-infected patients (66–67%) without any recent travel history were residents of the commune of Kobeni. However, in each commune of the department of Kobeni, malaria infection rates, defined as the number of PCR-confirmed malaria cases among febrile cases, were similar (range: 45–66%). The presence of semi-permanent rain-fed ponds, which allow *Anopheles* mosquitoes to breed, in Kobeni, Medbougou, and El Hassi communes may be one of the factors that explain high rates of malaria in the study area. Nonetheless, the low coverage of health facilities in the region, particularly in Kobeni department, is another factor that favours the movement of infected patients and probably the propagation of malaria parasites.

Individuals with travel history constitute an additional factor in disseminating malaria parasites. Although the present study did not confirm our initial hypothesis that Kobeni may be a major site where malaria-infected individuals from different countries transit and possibly spread malaria parasites, there is some evidence to suggest that malaria parasites are not only circulating in and around the commune of Kobeni but also being imported to the commune of Kobeni. Six ‘less developed’ rural-type communes around the commune of Kobeni are all characterized by high levels of malaria transmission during the rainy season. Malaria prevalence among patients with travel history (52%) and diagnosed with malaria in Kobeni and malaria prevalence in patients residing in Kobeni who had no travel history (60%) did not differ significantly (*P* > 0.05). Most patients reporting a recent travel history visited another malaria endemic area in Mauritania, while others, including a few foreigners, travelled to, or came from, another African country where malaria is known to be endemic and stable. In addition to this known factor, there is an unknown number of non-residents who get infected with malaria during their transit in Kobeni and are diagnosed once they return home. The real extent of the spread of malaria parasites to and from Kobeni is at present difficult to assess.

A unimodal pattern of malaria transmission characterizes this hotspot with malaria cases occurring mainly during and shortly after the rainy season with a peak in October. This pattern is characteristic of a sahelian zone where rainy season is usually short and irregular. Similar findings have been reported from other sahelian countries, such as Chad [34], Senegal [35], Niger [36], and Mali [37]. Association between malaria infection and the amount of rainfall was also observed in the present study. Indeed, the number of febrile patients who presented at the health centre and the number of patients with confirmed malaria during the transmission season of 2015 (annual rainfall, 448 mm) were about twice as high as those of 2016 (rainfall, 267 mm) and 2017 (rainfall, 263 mm). As expected from previous studies [32], very few malaria cases were observed during the dry season. Of 10 PCR-confirmed malaria cases, 5 were infected with *P. vivax* (1 with mixed *P. falciparum*-*P. vivax*) and 3 (including 2 with *P. vivax*) had a recent travel history to Nouakchott, where malaria transmission occurs throughout the year [1]. This suggests that many of the laboratory-confirmed malaria infections diagnosed during the dry season in Kobeni were probably due to *P. vivax* relapse and/or malaria imported from elsewhere. No major antimalarial intervention was implemented during this period in the region, with the exception of distribution of bed nets. Compared to 2015, there was a decrease in the number of febrile patients, with or without malaria, in 2016–2017. Based on these observations, it could be reasonably argued that the decrease in the amount of rainfall was probably unfavourable for productive breeding of malaria vectors and therefore for malaria transmission in 2016–2017.

Interventions to control malaria have not been well implemented in Kobeni. This observation is supported by several field observations. First, malaria prevalence has not diminished considerably between 2009 and 2010 and 2015–2017 [2]. Second, data on intermittent preventive treatment in pregnancy are not available. Third, a large majority of malaria-infected patients was treated with quinine, despite the national guideline that recommends artemisinin-based combination therapy (ACT). The efficacy of the first-line ACT, artesunate-amodiaquine, has been clinically proven in a study conducted in Kobeni in 2013 [31]. Fourth, although bed nets have been distributed, our study showed that close to one-third of febrile patients did not sleep under bed net. Patients who declared to have used insecticide-impregnated or non-impregnated bed nets were not protected from malaria, as compared to those who declared not to have slept under the bed net. These observations suggest that interventions are either not being implemented in the periphery of the country, in the case of ACT, or are not being properly implemented, in the case of bed nets. Mosquito bed nets, particularly long-lasting insecticide-treated nets (LLINs), have been proven to be highly effective in reducing malaria morbidity and mortality, particularly among children, when properly used [38, 39]. However, their effectiveness depends on several factors, including the house type and knowledge on the ability of bed nets to prevent malaria (i.e. educational level of the users). For instance, in our study population, the majority of febrile subjects attributed the usefulness of a bed net to the reduction of nuisance caused by mosquitoes rather than its capacity to prevent the bites of infective mosquitoes, to kill mosquitoes when it is insecticide impregnated, and as a consequence, to prevent from being infected by malaria parasites. Moreover, the level of insecticide resistance in *Anopheles arabiensis* is still very low (deltamethrin, 100% mortality; permethrin, 98.6% mortality) in Kobeni [9]. Therefore, there is a need to sensitize households on the sustained use of LLINs in order to optimize their role as an effective malaria control tool.

The earlier study conducted among symptomatic patients in Kobeni did not find any significant trend of malaria prevalence in relation with age groups [2]. In the present study, malaria affected more children between 5 and 9 years old than any other age groups among children. Although this finding may suggest that children of this age group are possibly more exposed to mosquito bites and/or that they have not developed an effective acquired immunity, a convincing explanation with verifiable evidence was not found in this study. Nonetheless, all age groups were affected by *P. falciparum* malaria in Kobeni and a low chemoprevention coverage (1.16%) among patients was observed. Therefore, interventions should target the most vulnerable populations, i.e. young children and pregnant women, but also older children and adults of all age in Kobeni. In this context, seasonal malaria chemoprevention (SMC) could be useful in preventing malaria episodes and associated parasitaemia and anaemia in children under 5 years and even in older children (up to 10 years) in the region [40]. Indeed, SMC was recommended in 2012 by the WHO for children aged 3–59 months living in areas with highly seasonal malaria transmission, as in the Sahelian region in Africa [41]. At present, SMC policy is being implemented in 12 countries in Africa’s sahelian sub-region [42], and assessment of its effectiveness in 3.2 million children showed that malaria prevalence was reduced by up to 65% [43]. In Mali, SMC was reported to reduce malaria infection and disease by more than 80% in children [44]. The implementation of SMC in Kobeni and surrounding communes needs population sensitization to obtain their active adherence to this novel strategy in Mauritania. Furthermore, as Kobeni is close to the border with Mali, a trans-border collaborative approach is required to control border malaria and counter the threat of spread of drug-resistant malaria.

One of the limitations of the present study is the recruitment of febrile patients in a health centre. Data analysed in the present study do not reflect the malaria situation in the general population in Kobeni in whom asymptomatic carriage of malaria parasites may occur in an unknown proportion of individuals. Studies designed to assess malaria prevalence in the general population would be required to further understand the dynamics of malaria transmission in southern Mauritania. Moreover, since the number of laboratory-confirmed malaria-infected patients was very low for two successive dry seasons in 2015 and 2016, patients were not recruited between January and July 2017. It is assumed that ‘missing data’ during this period in 2017 are similar to those observed in 2015 and 2016. In addition, it is known that malaria prevalence is correlated with the amount of rainfall. In southern Mauritania, the amount of rainfall varies from year to year. Therefore, the epidemiological pattern of malaria observed in Kobeni in 2015–2017 cannot be extrapolated to other time periods and places in southern Mauritania.

## Conclusions

Kobeni is highly endemic for malaria. Transmission is seasonal but intense. Given the context of border malaria, predominance of *P. falciparum* known to be chloroquine- and sulfadoxine-pyrimethamine resistant, and predominance of *Anopheles arabiensis*, the risk of spread of multidrug-resistant *P. falciparum* strains to other parts of the country and beyond the borders should not be underestimated. The stable level of malaria prevalence in Kobeni over the past 10 years, under-use of ACT, ineffective use or non-use of bed nets seem to be the main underlying causes of the lack of impact of interventions on malaria prevalence and apparent failure of control programme in Kobeni. More parasitological and entomological studies are required to assess malaria burden in this region. Interventions should be further reinforced in Kobeni, for example, by implementing SMC, to limit the risk of spread of malaria.

## Data Availability

All data generated or analysed during this study are included in this published article.

## Abbreviations

ACT: Artemisinin combination therapy
AL: Artesunate lumefantrine
ASAQ: Artesunate amodiaquine
CQ: Chloroquine
GMDP: Geometric mean of parasite density
LLINs: Long lasting insecticide nets
PCR: Polymerase chain reaction
RDT: Rapid diagnostic test
SMC: Seasonal malaria chemoprevention
SP: Sulfadoxine-pyrimethamine
